# Terpenoid Backbone Biosynthesis among Pig Hippocampal Pathways Impacted by Stressors

**DOI:** 10.3390/genes13050814

**Published:** 2022-05-02

**Authors:** Haley E. Rymut, Laurie A. Rund, Bruce R. Southey, Rodney W. Johnson, Sandra L. Rodriguez-Zas

**Affiliations:** 1 Department of Animal Sciences, University of Illinois at Urbana-Champaign, Urbana, IL 618012, USA; hrymut2@illinois.edu (H.E.R.); larund@illinois.edu (L.A.R.); southey@illinois.edu (B.R.S.); rwjohn@illinois.edu (R.W.J.); 2 Department of Statistics, University of Illinois at Urbana-Champaign, Urbana, IL 618012, USA

**Keywords:** RNAseq, maternal immune activation, weaning stress, double-hit hypothesis

## Abstract

Neurogenomic changes induced by maternal immune activation (MIA) during gestation and the social stress of weaning can alter brain plasticity in the hippocampus of offspring. The present study furthers the understanding of how these stressors impact hippocampus gene networks. The hippocampus transcriptome was profiled in pigs that were either exposed to MIA or not and were weaned or nursed. Overall, 1576 genes were differentially expressed (FDR-adjusted *p*-value < 0.05 and |log2 (fold change between pig groups)| > 1.2) in response to the main and interacting effects of MIA, weaning, and sex. Functional analysis identified 17 enriched immunological and neurological pathways in the Kyoto Encyclopedia of Genes and Genomes database. The enrichment of the terpenoid backbone biosynthesis pathway was characterized by genes under-expressed in MIA relative to non-MIA exposed, males relative to females, and weaned relative to nursed pigs. On the other hand, the enrichment of drug addiction pathways was characterized by gene over-expression in MIA relative to non-exposed pigs. Our results indicate that weaning and sex can modify the effects of MIA on the offspring hippocampus. This knowledge can aid in precise identification of molecular targets to reduce the prolonged effects of pre- and postnatal stressors.

## 1. Introduction

Weaning is a stressful event in an offspring’s life due to the combined effects of social and environmental changes [1,2]. Stress in weaned rodents and pigs arises from the offspring being removed from their mother and littermates, handling and transportation, new housing, and diet [1]. The increase in stress is also accompanied by alterations to hormonal and immunological changes and some of the processes are controlled by the hippocampus [3,4]. Relative to nursed rats, weaned rats show decreased levels of brain-derived neurotrophic factors in the hippocampus and prefrontal cortex [5] which are involved in learning and memory and are markers of neurodegenerative or neuropsychiatric diseases [6]. The hippocampus of weaned rats expressed altered levels of oxidants and had increased activity of superoxide dismutase, glutathione peroxidase, and lipid peroxidase, which could lead to altered learning and memory as well as increased anxious behaviors [7].

Another stressor that elicits behavioral, biomarker, and transcriptomic changes in young offspring is maternal immune activation (MIA). Environmental conditions or infection can induce MIA during gestation, which in turn affects cytokine signaling that can alter the brain and nervous system in the developing fetus [8,9]. A connection between MIA and behavioral disorders such as schizophrenia spectrum disorder (SSD) and autism spectrum disorder (ASD) have been reported in humans, rodents, and pigs [10,11,12].

The response to postnatal stress such as weaning can be affected by prenatal MIA [10,13,14,15]. Prenatal immune challenges can either sensitize or reduce the vulnerability of the offspring’s neurological networks to a secondary challenge and this “double hit” can have a prolonged impact on the offspring’s health and behavior. The double-hit hypothesis proposes that exposure to MIA elicits long-term immune and neural disruptions, leading to modification of the offspring’s response to a secondary immune challenge later in life [16,17]. A study of postnatal hypoxia-ischemia brain injury encompassing the hippocampus, cortex, and striatum demonstrated that the neuropathological damage from the second postnatal hit was more extensive in the offspring of mice exposed to MIA elicited by lipopolysaccharide (LPS) during gestation [16]. Studies have characterized the impact of pre- and postnatal stressors on behavior, peripheral biomarkers, hippocampus neurological damage, the amygdala transcriptome, and targeted immune and neuropeptide gene profiles [18,19,20,21,22]. Our study of the effects of a double hit of MIA and viral-like infection later in life uncovered sex-dependent changes in locomotor and other behaviors [20]. The behavioral changes elicited by MIA and secondary stress were related to changes in peripheral chemical and immune markers [19,23]. Additionally, the amygdala transcriptome of pigs exposed to MIA and weaning identified alterations in pathways associated with immune response, neuroactive ligand–receptor interaction, and glutamatergic processes [21,22]. A targeted comparison of the effect of MIA on neuropeptide isoforms highlighted distinct responses between the amygdala and the hippocampus including changes in hypocretin, peptide YY, and calcitonin-related polypeptide β [18].

Advances in brain transcriptomics are enabling the elucidation of gene networks associated with pre- and postnatal stressful factors. The unique response to the effects of MIA and a second stressor on the hippocampus, a plastic brain structure involved in learning and memory, remains partially understood [24]. The present study aims to advance previous research by offering insights into the repercussion of a double hit on the hippocampus transcriptome of females and males, which in turn may impact associated memory storage and retrieval processes. The main goal of the present study is to understand how the hippocampus gene networks are impacted by a double hit of weaning and MIA in pigs. The combined effects of (1) MIA stress due to infection, (2) weaning stress, (3) sex, and (4) the interactions between these effects were tested. A supporting objective of this study is to visualize gene networks by pathways of interest to aid in the understanding of the relationships between effects.

## 2. Materials and Methods

All experimental procedures followed previously published protocols [19]. The Illinois Institutional Animal Care and Use Committee (IACUC) at the University of Illinois approved that the animal studies are in compliance with the USDA Animal Welfare Act and the NIH Public Health Service Policy on the Humane Care and Use of Animals. Briefly, Camborough gilts inseminated with PIC boars were moved at gestation day 69 into individual disease containment chambers with a 12 h light/dark cycle. The gilts were fed a complete gestational diet and had ad libitum water access. Six gilts were inoculated with the porcine respiratory and reproductive virus (PRRSV; strain P129-BV) intranasally (5 mL of 1 × 10^5^ median tissue culture infectious dose diluted in sterile Dulbecco’s modified Eagle medium) while five gilts in the Control group were intranasally inoculated with 5 mL of the medium. All gilts were tested to confirm the presence or absence of PRRSV infection 1 week after exposure with a PCR test, and body temperature and feed intake for the two weeks following infection were also used to confirm illness.

Following previous protocols [19], farrowing was induced on gestation day 113 and thereafter the gilts were fed a nutritionally complete lactation diet. The pigs remained with the dam until 21 days of age, when approximately half of the pigs in each litter were weaned and the rest remained with the sow. Weaned pigs were housed in groups of four with ad libitum access to water and received a nutritionally complete diet for growing pigs. The experiment ended one day after weaning. The experimental design encompassed 48 pigs distributed across all eight groups representing MIA, weaning stress, and sex classes.

### 2.1. Hippocampal RNA Extraction and Sequencing

Pigs were anesthetized intramuscularly using a drug cocktail of telazol:ketamine:xylazine (50 mg of tiletamine; 50 mg of zolazepam) reconstituted with 2.5 mL ketamine (100 g/L) and 2.5 mL xylazine (100 g/L; Fort Dodge Animal Health, Fort Dodge, IA, USA) at a dose of 0.03 mL/kg body weight, following protocols [19] at 22 days of age. An intracardiac injection of sodium pentobarbital (86 mg/kg body weight, Fatal Plus, Vortech Pharmaceuticals, Dearborn, MI, USA) was used to euthanize pigs. The brains were removed, dissected, flash frozen on dry ice, and stored at −80 °C following published protocols [22]. From the hippocampi, an EZNA isolation kit (Omega Biotek, Norcross, GA, USA) was used to isolate RNA per the manufacturer’s instructions. An RNA integrity number of >7.3 was used as a cutoff for the samples to ensure low degradation. A TruSeq Stranded mRNAseq Sample Prep kit (Illumina Inc., San Diego, CA, USA) was used to prepare RNAseq libraries, and libraries were quantitated by qPCR and sequenced on one lane on a NovaSeq 6000 for 151 cycles from each end of the fragments using NovaSeq S4 reagent kit. The bcl2fastq v2.20 conversion software was used to produce and demultiplex FASTQ files.

### 2.2. RNA Sequence Mapping and Differential Expression Analysis

Assessment of read quality found a minimum Phred score of 35 across all read positions using FASTQC [25], meaning no reads needed to be trimmed for poor quality. Paired-end reads were aligned to the *Sus scrofa* transcriptome (version *S. scrofa* 11.1; [26]) and quantified using kallisto v0.43.0 [27] with default settings. Normalized gene expression values were described using a generalized linear model encompassing the effects of the MIA group, weaning stress, sex, and the two-way interactions. Genes supported by more than 5 transcripts per million RNA molecules by each weaning–MIA–sex combination were analyzed for differential gene expression using edgeR (v3.14.0) in the R v3.3.1 environment [28]. The cutoff for differential expression was set to a False Discovery Rate (FDR) [29]-adjusted *p*-value < 0.05 and a log2 (fold change between pig groups) > |1.2| to highlight strong trends.

### 2.3. Functional Enrichment, Network Inference and Transcriptional Factor Analysis

Identification of over-represented Kyoto Encyclopedia of Genes and Genomes (KEGG) pathways [30,31] among all genes analyzed for each effect was performed using Gene Set Enrichment Analysis (GSEA) with WebGESTALT [32]. The GSEA used information from all genes analyzed, ranked from the most over-expressed to the most under-expressed to identify over-represented pathways. The normalized enrichment score (NES) of the pathways was calculated using the maximum deviation of the cumulative sum based on the signed fold change of the ranked genes divided by the average of the permutated enrichment scores. For the enrichment analysis, the *Sus scrofa* genome was selected and enriched pathways with a minimum of five genes and a maximum of 2000 genes were evaluated. Statistical significance was determined by calculating the FDR-adjusted *p*-value from 1000 permutations. A cutoff for differential expression of FDR-adjusted *p*-value 0.05 and NES > 1.5 or NES < 1.5 was considered to highlight enriched pathways.

To understand the potential changes in the relationship between genes elicited by MIA in either weaning group, gene networks-based gene expression profiles were constructed with Cytoscape v3.8.1 [33] and STRING database [34]. In the networks, the color of the gene reflects the sign of the fold change, where red denotes under-expression and green denotes over-expression.

## 3. Results

### 3.1. Hippocampal Gene Expression Profiles

Sequencing produced 6,116,300,000 reads across all 48 libraries. The median number of reads per pig was consistent across different groupings, with 116,380,000 reads for PRRSV-exposed pigs; 120,500,000 reads for Control pigs; 119,360,000 reads for weaned pigs; 117,850,000 reads for nursed pigs; 122,830,000 reads for female pigs; and 116,600,000 reads for male pigs. The median number of reads mapped per pig was 95,570,000. These reads were mapped to 18,295 genes with >5 mapped read counts per group.

At a FDR-adjusted *p*-value < 0.05 and a |log2 (fold change between pig groups)| > 1.2, differential expression was detected on 173 genes for a MIA-by-weaning effect, 481 genes for a MIA-by-sex effect, 175 genes for a weaning-by-sex effect, 545 genes for a MIA effect, 55 genes for a weaning effect, and 147 genes for a sex effect.

### 3.2. The Effects of Weaning, Maternal Immune Activation and Sex on Hippocampal Pathways

Table 1 depicts the KEGG pathways enriched in at least one of the interactions or main effects with an FDR-adjusted *p*-value < 0.05 and a NES > |1.5|. The sign of the NES values (Table 1) offers insights into the predominant differential expression pattern in the pathway elicited by MIA, weaning stress, or sex. Overall, the GSEA analysis identified 17 enriched immune- and neurologically related pathways. Of the pathways, 8 were enriched among genes that had an MIA effect, 8 pathways were enriched among genes that had a weaning stress effect, 10 pathways were enriched among genes that had a sex effect, 9 pathways were enriched among genes that had an MIA-by-weaning interaction effect, and 13 pathways were enriched among genes that had an MIA-by-sex interaction effect. No pathways were enriched with interaction between sex and weaning.

Metabolic pathways were over-expressed in Control compared to MIA. The terpenoid backbone biosynthesis pathway (ssc00900) was enriched among genes over-expressed in Control compared to MIA pigs, over-expressed in male relative to female pigs, and among genes over-expressed in nursed relative to weaned pigs. The other enriched metabolic pathway, the oxidative phosphorylation pathway (ssc00190), was enriched among genes over-expressed in Control compared to MIA pigs and is under-expressed in males relative to females.

Nervous system pathways showed a consistent enrichment pattern characterized by genes over-expressed in MIA relative to Control pigs. This consistency was expected due to the shared genes in the dopaminergic, nicotine, cocaine, morphine, GABAergic, and cholinergic pathways (ssc05033, ssc05032, ssc04727, and ssc04725). These pathways were not enriched directly by weaning stress or sex, although three nervous system pathways showed a significant interaction between MIA and sex.

While pigs were not directly exposed to PRRSV, several immune-related pathways were enriched involving an interaction with MIA. All nine immune-response pathways were enriched with the interaction of MIA and sex and MIA and weaning. However, none of these pathways were enriched by MIA alone, indicating that MIA influenced the effects of weaning and sex on these pathways. The consistency between these immune pathways is due to the shared genes.

### 3.3. The Effects of Weaning, Maternal Immune Activation and Sex on Gene Expression Profiles

The detailed lists of genes that had MIA, weaning, or sex effects (FDR-adjusted *p*-value < 0.05, and |log2 (fold change between pig groups)| > 2) irrespective of pathway annotation are presented in Supplementary Tables S1–S3. Gene patterns that had a significant FDR-adjusted *p*-value < 0.05, and a |log2 (fold change between pig groups)| > 1.2 for the interactions or main effect of MIA, weaning, or sex and correspond to a pathway category enriched at a FDR-adjusted *p*-value < 0.05 and |NES| > 1.5 are listed in Table 2.

Considering all genes with a significant (FDR-adjusted *p*-value < 0.05 and |log2 (fold change between pig groups)| > 1.2) effect, approximately 70% of these genes affected by MIA were under-expressed in MIA compared to Control pigs. Notable gene families included three dopamine receptor genes, four phosphodiesterase genes, six swine leukocyte antigen (SLA) genes and three solute carrier family genes. Likewise, approximately 72% of the genes that had a weaning effect were under-expressed in weaned relative to nursed pigs. The majority of genes presenting sex effects (63%) were under-expressed in males relative to females. Most of the genes with a significant interaction involving MIA also were significant for the main MIA effect, indicating a direct influence of MIA on these genes. The SLA genes, associated with the swine MHC complex class II, exhibited interactions with MIA and were significant for sex and weaning. These were consistent with the related immune pathways that indicated sex and weaning effects that were magnified by MIA.

### 3.4. The Effects of Weaning, Maternal Immune Activation and Sex on Gene Networks in the Hippocampus

**Figure 1:**
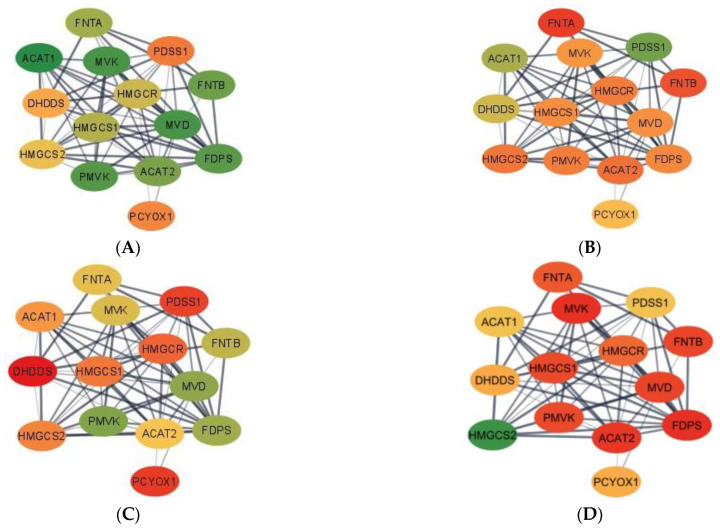

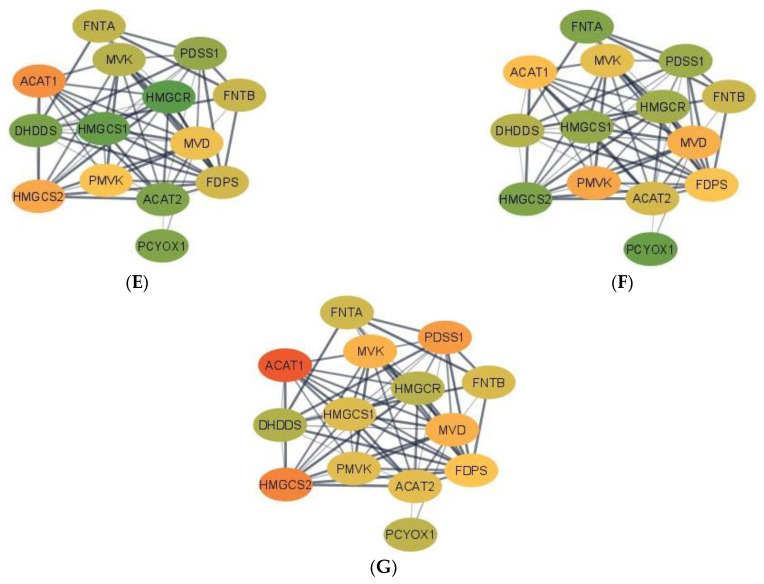
Gene networks for the terpenoid backbone biosynthesis pathway in the hippocampus developed using Cytoscape and the STRING database. Nodes represent genes and edges represent database connections. The node color ranges from red that signifies a −2.0 log2 (fold change), yellow indicates no fold change, and green indicates a 2.0 log2 (fold change), with colors in between indicating a spectrum. Changes correspond to seven pig groups compared to the baseline Control nursed males. The seven pig groups are: (**A**)—Control nursed females, (**B**)—MIA nursed females, (**C**)—Control weaned females, (**D**)—MIA weaned females, (**E**)—MIA nursed males, (**F**)—Control weaned males, and (**G**)—MIA weaned males compared to Control nursed males.

**Figure 2:**
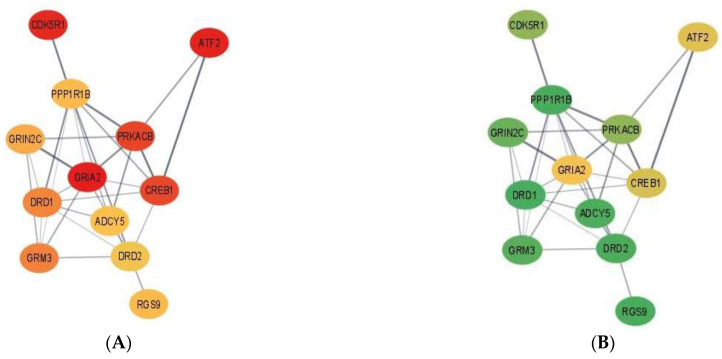

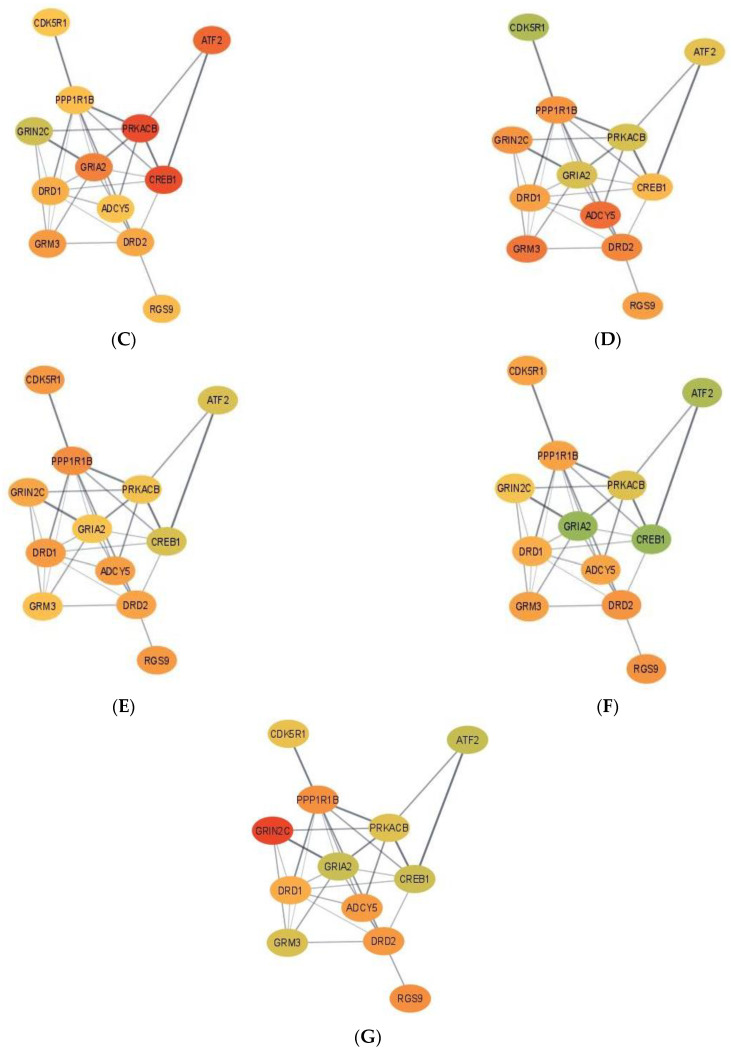
Gene networks for the cocaine addiction pathway in the hippocampus developed using Cytoscape and the STRING database. Nodes represent genes and edges represent database connections. The node color ranges from red that signifies a −2.0 log2 (fold change), yellow indicates no fold change, and green indicates a 2.0 log2 (fold change), with colors in between indicating a spectrum. Changes correspond to seven pig groups compared to the baseline Control nursed males. The seven pig groups are: (**A**)—Control nursed females, (**B**)—MIA nursed females, (**C**)—Control weaned females, (**D**)—MIA weaned females, (**E**)—MIA nursed males, (**F**)—Control weaned males, and (**G**)—MIA weaned males compared to Control nursed males.

Complementing pathways and gene patterns, the study of gene interactions provided insights into the potential impact of the first and second stressors on the relationships between genes. Gene networks depicting the connection between gene products based on the PPI BIOGRID database and integrating the differential expression between pig groups were depicted for enriched pathways including less than 15 gene nodes to facilitate visualization. These pathways included terpenoid backbone biosynthesis (Figure 1) and cocaine addiction (Figure 2). The differential expression was measured in terms of log2 (fold change) between each one of the seven pig groups simultaneously denoted by the MIA, weaning, and sex levels relative to a baseline pig group set to Control nursed males. The color scheme of the nodes ranges from green to yellow and to red representing positive, approximately null, and negative fold changes, respectively. The seven contrasts depicted in the networks correspond to (A) Control nursed females, (B) MIA nursed females, (C) Control weaned females, (D) MIA weaned females, (E) MIA nursed males, (F) Control weaned males, and (G) MIA weaned males with each group compared to Control nursed males.

Networks for the terpenoid backbone biosynthesis pathway for the seven pig group contrasts are presented in Figure 1. Overall, most genes in the terpenoid backbone biosynthesis pathway were under-expressed in MIA weaned females when compared to the Control nursed males (Figure 1D). On the other hand, most genes were over-expressed in Control nursed females when compared to the Control nursed males illustrating the effect of sex on the gene expression (Figure 1A). The consideration of both contrasts indicates that the highest differences in expression for the terpenoid backbone biosynthesis pathway were over-expression in Control nursed relative to MIA weaned females. Fold-change values are relative to the baseline Control nursed males and are presented in Supplementary Table S4.

In the terpenoid backbone biosynthesis pathway, acetyl-CoA acetyltransferase 1 (ACAT1) and acetyl-CoA acetyltransferase 2 (ACAT2) presented common profiles in some pig groups and distinct profiles in others. For example, ACAT1 and ACAT2 had a high positive fold change when comparing Control nursed females to their male counterparts, while the highest negative fold change for ACAT1 was detected in MIA weaned males and in MIA weaned females for ACAT2. The dehydrodolichyl diphosphate synthase subunit (DHDDS) had the highest positive fold change in MIA nursed males and the highest negative fold change in Control weaned females. Farnesyl diphosphate synthase (FDPS) had the highest positive fold change in Control nursed females and the highest negative fold change for MIA weaned females. Farnesyltransferase, CAAX Box, α (FNTA) had the highest positive fold change in Control weaned males and the highest negative fold change in MIA nursed females. In contrast, farnesyltransferase, CAAX Box, β (FNTB) had the highest positive fold change in Control weaned females and the highest negative fold or MIA weaned females. 3-hydroxy-3-methylglutaryl-CoA reductase (HMGCR) had the highest positive fold change in MIA nursed males and the highest negative fold change in Control weaned females. 3-hydroxy-3-methylflutaryl-CoA synthase 1 (HMGCS1) had the highest positive fold change in MIA nursed males and the highest negative fold change in MIA weaned females. However, 3-hydroxy-3-methylflutaryl-CoA synthase 2 (HMGCS2) had the highest positive fold change in MIA weaned females and the highest negative fold change in MIA nursed females. Mevalonate diphosphate decarboxylase (MVD) had the highest positive fold change in Control nursed females and the highest negative fold change in MIA weaned females. Mevalonate kinase (MVK) had the highest positive fold change in Control nursed females and the highest negative fold change in MIA weaned females. Prenylcysteine oxidase 1 (PCYOX1) had the highest positive fold change in Control weaned males and the highest negative fold change in Control weaned female. Decaprenyl diphosphate synapse subunit 1 (PDSS1) had the highest positive fold change in MIA nursed females and the highest negative fold change in Control weaned females. Phosphomevalonate kinase (PMVK) had the highest positive fold change in Control nursed females and the highest negative fold change in MIA weaned females relative to Control nursed males.

Figure 2 depicts the networks for the cocaine addiction pathway and fold-change values are relative to the baseline Control nursed males and are presented in Table S5. Overall, most genes in the cocaine addiction pathway were under-expressed in Control nursed females relative to Control nursed males indicating that under no first or second stressors, there is an effect of sex on cocaine pathway genes (Figure 2A). On the other hand, most genes in the cocaine addiction pathway were over-expressed in MIA nursed females compared to Control nursed males (Figure 2B).

Within the cocaine addiction pathway, the highest positive fold change occurred in Control weaned females for 9 of the 12 genes: adenylate cyclase 5 (ADCY5), cyclin-dependent kinase 5 regulatory subunit 1 (CDK5R1), dopamine receptor D1 (DRD1), dopamine receptor D2 (DRD2), glutamate ionotropic receptor NMDA-type subunit 2C (GRIN2C), glutamate metabotropic receptor 3 (GRM3), protein kinase CAMP-activated catalytic subunit β (PRKACB), PPP1R1B, and RGS9. The remaining 3 genes, iron-regulated transcriptional activator (ATF2), CAMP responsive element binding protein 1 (CREB1) and glutamate ionotropic receptor AMPA-type subunit 2 (GRIA2), had the highest positive fold change in MIA weaned females. The lowest negative fold change varied across genes but the lowest negative fold change in ATF2, CDK5R1, CREB1, DRD1, GRIA2, and PRKACB occurred in Control nursed females. The lowest negative fold change in ADCY5, DRD2, and GRM3 occurred in MIA nursed females.

## 4. Discussion

### 4.1. Hippocampal Pathways and Genes Affected by Maternal Immune Activation, Weaning and Sex Interactions

The functional analysis identified 17 KEGG pathways enriched among gene profiles impacted by MIA, weaning, sex, or the interactions among the factors (Table 1). Overall, the expression of 574 genes was significantly affected (FDR-adjusted *p*-value < 0.05 and |log2 (fold change between pig groups)| > 1.2) by at least one of the factors studied including MIA, weaning, and sex acting individually or through interactions. The enriched pathways can be grouped into three categories, (i) immune system (e.g., antigen, infection, and inflammation processes), (ii) nervous system (e.g., addiction and synapse pathways), and (iii) metabolic system (e.g., steroid-associated terpenoid synthesis and oxidative phosphorylation).

#### 4.1.1. Enrichment of Immunological and Neurological Pathways

The enrichment of immunological and neurological pathways may be related to the MIA effects on neurodevelopment and weaning stress. Associations between antigen-processing and -presentation molecules and the pathway and MIA conditions or disorders such as ASD and SSD have been reported in humans and rodents [35,36,37]. Likewise, our study of the effects of MIA on pig amygdala also identified the enrichment of immune pathways [22].

The positive NES of immune system pathways such as herpes simplex infection in the MIA interactions with weaning and sex, and the negative NES for MIA (albeit not reaching significance threshold) are associated with over-expression in MIA pigs, specifically in nursed females.

Similarly, the negative NES for immune system categories indicates under-expression of the pathway genes associated with weaning. The most extreme genes under-expressed in weaning relative to nursed pigs (log2 (fold change) < −1.5) included swine leukocyte antigen—MHC class II DR α (SLA-DRA), cathepsin L (CTSL), and swine leukocyte antigen—MHC class II DQ α 1 (SLA-DQA1). Our findings are also consistent with reports of under-expression of antigen presentation transcripts in the cord blood of children exposed to early-life stress [38].

Enriched immune categories for a sex effect were observed with the negative NES. This effect of sex alone or interacting with MIA on the immune system transcriptome of the hippocampus aligns with reports of the sex-dependent effects of MIA on the immune-related transcriptome in rodent placenta [37] and fetal brains [39].

The simultaneous consideration of primary and secondary stressors and sex effects on the genes annotated to immune system pathways enabled us to uncover the most extreme over-expression of swine MHC complex class II genes SLA-DRA, SLA-DQA1, swine leukocyte antigen MHC class II DQ haplotype C β chain (SLA-DQB1), and swine leukocyte antigen MHC class II DM α (SLA-DMA) in the hippocampus from MIA weaned males relative to Control nursed males. This notable result highlights the synergistic action of primary and secondary stressors and the heightened regulation of genes associated with immune response. Other genes such as swine leukocyte antigen MHC class II DR β 1 (SLA-DRB1) were over-expressed in MIA relative to Control males, irrespectively of second stressor level, suggesting a dominating effect of the primary stressor, whereas these previous genes were highly under-expressed in MIA weaned females relative to Control nursed males. This finding accentuates the sex-dependent nature of the interaction between primary and secondary stressors on the pathways of the immune system. The enrichment of immune system pathways is consistent with detected changes in peripheral immune biomarkers, including interleukin 1β, interleukin 8, interleukin 2, interferon γ, globulin, and total protein, associated with MIA and secondary stressors in 3- and 8-week-old pigs [21].

#### 4.1.2. Enrichment of Nervous System Pathways

The strongest enrichment of nervous system categories corresponded to the effect of MIA. Enriched categories include addictive pathways that share genes (i.e., cocaine, morphine, and nicotine), and GABAergic, cholinergic, and dopaminergic synapse pathways (Table 1). The under-expressed genes (FDR-adjusted *p*-value < 0.05 and log2 (fold change) < −1.2) included the GABAergic synapse pathway genes of GAD2 and GABRA4 and the cholinergic synapse pathway genes of SLC5A7, CAMK4, and CHRM4. The most extreme differentially expressed genes were annotated to the dopaminergic pathway and included DRD1, DRD2, DRD3, PPP1R1B, and GNAL (−4.4 < log2 (fold change) < −1.8). Alterations in dopamine pathways have been correlated to increased risk of drug addiction in ASD patients [40]. Evidence of the brain-region dependency of the effect of MIA on the molecular systems is that the previous pathways were not enriched in the amygdala of MIA-exposed pigs [21].

The negative NES of the neurological categories for the MIA effect indicates gene over-expression in the hippocampus of MIA relative to Control pigs. Our finding is consistent with reports that Poly(I:C) elicited MIA in rodents alters GABAergic polarity and circuity in the hippocampus [41]. Dysregulated GABAergic polarity is a marker of developmental brain disorders, and, consistent with the observed over-expression of genes in the pig GABAergic pathway, MIA abolishes the protective inhibitory action of GABA at birth in hippocampal neurons in rodent models of ASD [42]. A study of hippocampal neurons in mice exposed to Poly(I:C) during gestation uncovered that GABAergic interneurons are highly impacted by MIA [43]. Dysfunction of the cholinergic system has been linked to MIA disorders including ASD and SSD [44]. The over-expression of cholinergic synapse genes in MIA-exposed pigs (i.e., negative enrichment score) is in agreement with the increase in cholinergic system gene expression in mouse fetal brain after Poly(I:C)-elicited MIA [44].

#### 4.1.3. Enrichment of Metabolic Pathways

Significant enrichment of the oxidative phosphorylation pathway among the genes presenting an MIA effect is aligned with studies that linked this pathway to MIA and neurodevelopmental disorders [45]. The positive enrichment score of the oxidative phosphorylation pathway indicates that most differentially expressed genes were under-expressed in MIA relative to Control pigs. The most extreme gene was ATPase H+ Transporting V1 Subunit C2 (ATP6V1C2) (log2 (fold change) > 1.7; Table 2) that encodes a component of ATPase. Reduced expression of ATPase genes is associated with neurodevelopmental and neurodegenerative diseases [46]. Consistent with our findings, genes associated with mitochondrial oxidative phosphorylation were under-expressed in MIA-exposed mouse offspring [45]. In contrast, the effect of MIA on the oxidative phosphorylation pathway in the amygdala was not significant [21], suggesting that MIA may have differing effects in each brain region.

### 4.2. Hippocampal Gene Networks Affected by Maternal Immune Activation, Weaning and Sex Interactions

Among the enriched pathways encompassing genes impacted by the effects of MIA, weaning, and sex, the networks of gene expression profiles for two pathways were further investigated. The terpenoid backbone biosynthesis and cocaine addiction pathways offered complementary understanding of two major functional structures, the metabolic, and nervous systems impacted by primary and secondary stressors. Notably, these pathways were not enriched in the study of the amygdala transcriptome, suggesting a specific participation in processes related to the role of the hippocampus [22].

#### 4.2.1. Terpenoid Backbone Biosynthesis Pathway

Members of the terpenoid backbone biosynthesis pathway have been proposed as a treatment method for MIA-associated ASD and to offer protection against neurodegenerative disorders [47]. Terpenoid treatments can diminish hippocampus neuronal losses and improve memory deficits [48].

Visualization of the gene expression patterns in the terpenoid backbone biosynthesis pathway further advanced the understanding of the established therapeutic effects of the pathway molecules on disorders associated with the hippocampus function. Broadly, many genes were over-expressed in the hippocampus of Control nursed females relative to MIA weaned females (Table 2, Figure 1). This finding suggests that MIA and weaning stresses may act together in a most extreme manner in females.

Additionally, most genes in the terpenoid backbone biosynthesis pathway were over-expressed in Control nursed females relative to Control nursed males, and this finding suggest a significant sex effect under baseline conditions devoid of primary or secondary stressors. As a colorway of the previous patterns, most genes tended to be under-expressed in MIA weaned females relative to Control nursed males. The previous findings suggest that in the terpenoid backbone biosynthesis pathway, MIA and weaning stresses may act together in a most extreme manner in females while sex differences can be detected under baseline conditions devoid of primary or secondary stressors.

A notable finding in the terpenoid backbone biosynthesis pathway was the range of patterns among homolog genes. While both FNT subunit genes (FNTA and FNTB) presented a similar profile across pig groups, two genes coding acetyl-CoA C-acetyltransferase enzymes (ACAT1 and ACAT2) have different effects in several MIA-weaning-sex groups. Inconsistent patterns across pig groups were also observed in two genes coding for the same hydroxymethylglutaryl-CoA synthase enzyme (HMGS1, and HMGS2). However, genes HMGCR and HMGCS1 have very similar pattern, whereas one gene codes for hydroxymethylglutaryl-CoA reductase and the other for hydroxymethylglutaryl-CoA synthetase. HMGCS2 was over-expressed in all groups when compared to Control nursed males, with MIA weaned females having the greatest fold change (Figure 1). HMGCS2 is involved in lipid-derived energy creation and fatty acid breakdown [49], with long-chain fatty acids offering neuron protection and are used as therapy against neurological disorders such as ASD [50].

#### 4.2.2. Addiction Pathways

The relation between MIA and addiction pathways detected in the present study may be associated with reports of associations between MIA and drug addiction behaviors. Environmental challenges during gestation may hinder brain development and these events have been associated with higher incidence of MIA-associated disorders such as SSD and addictive behaviors [51]. A study of mice challenged with Poly(I:C) during gestation demonstrated that MIA was associated with amphetamine-induced behavioral sensitization, conditional place preference, and cross-sensitization to cocaine [51]. Likewise, LPS-elicited MIA and peri-pubertal stress influenced cocaine addiction-like behaviors and striatal transcriptome in rats [52]. In the network of genes annotated to the cocaine pathway (also in the nicotine addiction pathway), ADCY5, DRD1, DRD2, PPP1R1B, and protein kinase C β (PRKCB) were over-expressed in Control nursed males relative to all pig groups with exception of Control weaned females (Figure 2). This network profile highlights the effect of MIA lowering the levels of the dopamine receptors, which in turn impacts neurotransmission. The similar pattern exhibited by Control weaned males suggests that the secondary stress can also have a detrimental and sex-dependent effect on neurotransmission. Lastly, sex effects on gene expression profiles have been reported in addiction pathways [53]. The differences between sexes in the expression of cocaine addiction genes such as CREB1 and protein kinases such as PRKACB have been previously reported in mice under other experimental conditions [54].

Overall, the gene expression was lower in Control nursed females relative to Control nursed males in the addiction pathway. This result indicates that in the absence of first or second stressors, females have lower expression of cocaine pathway genes. In addition, genes were over-expressed in MIA nursed females relative to Control nursed males for many genes in the pathway. Interestingly, the hippocampus gene expression in females was lower than males under no stress. However, in the presence of MIA, gene expression was higher in females than in males. This result indicates that MIA elicits over-expression of cocaine addiction pathway genes in females, but this effect was not observed in males.

While Control nursed females had the lowest expression of genes in the cocaine addiction pathway relative to Control nursed males, the presence of MIA or weaning diminished the previous sex difference. Therefore, our results suggest that a first or second stressor can attenuate the difference in expression between sexes in cocaine addiction pathway genes. The lowest overall gene expression difference was observed between MIA weaned males and Control nursed males, suggesting that the effect of MIA could compensate or cancel the effect of weaning in males for genes in the cocaine addiction pathway. In general terms, Control weaned males were the only group presenting gene over-expression relative to Control nursed males. This unique pattern among pig groups suggests that the second stressor of weaning can elicit a particular expression profile. Both network extremes demonstrate the impact of sex, alone or interacting with MIA, on molecular pathways in the hippocampus.

## 5. Conclusions

The present study evaluated the effects of MIA, the secondary social stress of weaning, and sex on the hippocampus transcriptome of male and female pigs. Our brain transcriptomic study supports the double-hit hypothesis examined in studies of behavioral disorders associated with SSD and ASD. The relationship between MIA and weaning was strongest in nine immunological pathways including the antigen-processing and -presentation and the Rheumatoid arthritis pathways. Genes annotated to these immunological pathways (e.g., SLA-DQA1 and SLA-DRA) presented consistent expression patterns across the stress levels studied.

A three-way interaction between sex, maternal immune activation, and weaning stress was detected in the terpenoid backbone biosynthesis and cocaine addiction pathways. Among the genes presenting distinct profiles between sexes across stress groups were HMGCS2, DRD1, and PPP1R1B. Through the identification of hippocampal genes and pathways, therapeutic treatments can be developed to counteract the effects of weaning and MIA. Our results highlight the need for further exploration into the therapeutic effects of terpenoids in double-hit conditions.

## Figures and Tables

**Table 1:** Hippocampal pathways enriched by at least one factor including interactions (x) or the main effects of weaning (W), maternal immune activation (M), and sex (S) at a FDR-adjusted *p*-value < 0.05 and a normalized enrichment score |NES| > 1.5.

KEGG Pathway ^1^	MxS		M ^2^		S		WxM		W	
	N ^3^	FDR	N	FDR	N	FDR	N	FDR	N	FDR
*Metabolism*										
Terpenoid backb.	2.0	3 × 10^−4^	1.9	4 × 10^−2^	−1.7	5 × 10^−2^			−2.0	5 × 10^−3^
Oxidative phosph.			1.9	3 × 10^−2^	−1.7	3 × 10^−2^				
*Nervous system*										
Dopaminer synap.	−1.8	2 × 10^−2^	−1.8	4 × 10^−2^						
Nicotine addiction			−1.8	4 × 10^−2^						
Cocaine addict.	−1.9	1 × 10^−2^	−2.0	7 × 10^−3^						
Morphine addict.	−1.8	4 × 10^−2^	−2.1	1 × 10^−6^						
GABAergic synap.			−1.9	2 × 10^−2^						
Cholinergic synap.			−1.8	5 × 10^−2^						
*Immune system*										
Allograft rejection	2.2	1 × 10^−6^			−2.4	1 × 10^−6^	2.4	1 × 10^−6^	−1.8	7 × 10^−3^
Antigen processing	2.6	1 × 10^−6^			−2.7	1 × 10^−6^	2.6	1 × 10^−6^	−2.1	4 × 10^−3^
Asthma	2.0	3 × 10^−4^			−2.2	2 × 10^−4^	2.2	1 × 10^−4^	−1.8	5 × 10^−3^
Autoimm. thyroid	2.2	1 × 10^−6^			−2.3	1 × 10^−6^	2.5	1 × 10^−6^	−1.8	5 × 10^−3^
Herpes infection	2.4	1 × 10^−6^					2.1	3 × 10^−3^		
Staphyloco. infect.	2.2	1 × 10^−6^			−2.6	1 × 10^−6^	2.4	1 × 10^−6^	−2.4	1 × 10^−6^
Systemic lupus.	2.1	1 × 10^−6^			−2.1	2 × 10^−4^	1.9	1 × 10^−2^	−1.9	2 × 10^−2^
Rheumatoid arthr.	2.0	4 × 10^−4^			−2.1	3 × 10^−3^	2.2	1 × 10^−6^	−1.9	2 × 10^−2^
Graft-versus-host	2.3	1 × 10^−6^			−2.4	1 × 10^−6^	2.3	1 × 10^−6^		

^1^ KEGG = Kyoto Encyclopedia of Genes and Genomes pathway. Ssc00900 = Terpenoid backbone biosynthesis.; ssc00190 = oxidative phosphorylation; ssc04728 = dopaminergic synapse; ssc05033 = Nicotine addiction; ssc05030 = cocaine addiction; ssc05032 = morphine addiction; ssc04727 = GABAergic synapse; ssc04725 = cholinergic synapse; ssc05330 = allograft rejection; ssc04612 = antigen processing and presentation; ssc05310 = asthma disease; ssc05320 = autoimmune thyroid disease; ssc05168 = herpes simplex infection; ssc05150 = Staphylococcus aureus infection; ssc05322 = systemic lupus erythema.; ssc05323 = rheumatoid arthritis disease; ssc05332 = graft-versus-host disease. ^2^ Normalized enrichment score (NES): MxS = MIA-by-sex interaction effect, where NES > 0 (NES < 0) denotes gene over-expression (under-expression) in Control females; M = maternal immune activation effect, where NES > 0 (NES < 0) denotes gene over-expression (under-expression) in Control relative to MIA pigs; S = sex effect, where NES > 0 (NES < 0) denotes gene over-expression (under-expression) in males relative of females; WxM = weaning-by-MIA interaction effect, where NES > 0 (NES < 0) denotes gene over-expression (under-expression) in Control weaned pigs; W = weaning effect, where NES > 0 (NES < 0) denotes gene over-expression (under-expression) in weaned relative to nursed pigs. ^3^ N = normalized enrichment score (NES); FDR = FDR-adjusted *p*-value.

**Table 2:** Log2 (fold change) and False Discovery Rate-adjusted *p*-value (FDR) of genes in the hippocampus that presented at least one significant (FDR *p*-value < 0.05 and a |log2 (fold change between pig groups)| > 1.2) interaction or main effect of weaning (W), maternal immune activation (M), and sex (S) in the pathways enriched at a FDR-adjusted *p*-value < 0.05 and a normalized enrichment score |NES| > 1.5.

Gene Symbol	MxS ^1^	WxM	M		S		W	
	FDR	FDR	LogFC	FDR	LogFC	FDR	LogFC	FDR
Metabolic pathway								
ATP6V1C2	5 × 10^−3^		1.68	4 × 10^−5^				
HMGCS2	4 × 10^−2^				−1.38	1 × 10^−2^		
Nervous system pathway								
ADCY5	8 × 10^−4^							
CAMK4			−1.30	1 × 10^−3^				
CHRM4			−1.28	2 × 10^−3^				
DRD1	6 × 10^−45^		−3.96	3 × 10^−29^				
DRD2	7 × 10^−81^		−4.37	3 × 10^−34^				
DRD3	4 × 10^−16^		−2.53	5 × 10^−12^				
GABRA4	5 × 10^−2^		−1.25	3 × 10^−3^				
GAD2			−1.53	7 × 10^−5^				
GNAL	4 × 10^−15^		−1.75	2 × 10^−6^				
GNG7	2 × 10^−4^							
GRK5	8 × 10^−3^							
PDE10A	2 × 10^−6^		−1.73	4 × 10^−6^				
PDE1B	2 × 10^−15^		−2.10	5 × 10^−9^				
PDE7B	2 × 10^−7^		−1.56	5 × 10^−5^				
PDE8B	7 × 10^−3^		−1.43	2 × 10^−4^				
PPP1R1B	5 × 10^−47^		−3.15	1 × 10^−19^				
RGS9	8 × 10^−30^		−2.63	7 × 10^−14^				
SLC17A6			−1.51	1 × 10^−4^				
SLC32A1	4 × 10^−5^							
SLC5A7			−2.12	1 × 10^−8^				
Immune system pathway								
CCL2	9 × 10^−4^							
CD74	9 × 10^−6^	5 × 10^−3^						
CTSL	1 × 10^−9^	9 × 10^−3^			−1.57	4 × 10^−4^	−1.63	4 × 10^−4^
DDX58	4 × 10^−2^							
SLA-5	4 × 10^−4^							
SLA-DMA	2 × 10^−2^							
SLA-DQA1	2 × 10^−7^	8 × 10^−5^					−1.57	1 × 10^−4^
SLA-DQB1	4 × 10^−4^							
SLA-DRA	3 × 10^−8^	2 × 10^−4^			−1.45	1 × 10^−3^	−1.68	3 × 10^−4^
SLA-DRB1	9 × 10^−7^							

^1^ False Discovery Rate-adjusted *p*-value (FDR) and log2-transformed fold change between pig groups (LogFC). MxS = MIA-by-sex interaction effect; WxM = weaning-by-MIA interaction effect; M = maternal immune activation effect, where LogFC > 0 (LogFC < 0) denotes gene over-expression (under-expression) in Control relative to MIA pigs; S = sex effect, where LogFC > 0 (LogFC < 0) denotes gene over-expression (under-expression) in males relative of females; W = weaning effect, where LogFC > 0 (LogFC < 0) denotes gene over-expression (under-expression) in weaned relative to nursed pigs.

## Data Availability

Data available from corresponding author on request.

## Supplementary Materials

The following supporting information is available online at https://www.mdpi.com/article/10.3390/genes13050814/s1, Table S1: Log (fold change), False Discovery Rate-adjusted *p*-value (FDR), and raw *p*-values of all genes that presented a significant (FDR *p*-value < 0.05 and |log2 (fold change between pig groups)| > 1.2) main effect of maternal immune activation; Table S2: Log (fold change), False Discovery Rate-adjusted *p*-value (FDR), and raw *p*-values of all genes that presented a significant (FDR *p*-value < 0.05 and |log2 (fold change between pig groups)| > 1.2) main effect of weaning; Table S3: Log (fold change), False Discovery Rate-adjusted *p*-value (FDR), and raw *p*-values of all genes that presented a significant (FDR *p*-value < 0.05 and |log2 (fold change between pig groups)| > 1.2) main effect of sex; Table S4: Log (fold change) of pairwise contrasts of genes in the terpenoid backbone biosynthesis pathway; Table S5: Log (fold change) of pairwise contrasts of genes in the cocaine addiction pathway.
