# Supplementary material for: Terpenoid Backbone Biosynthesis among Pig Hippocampal Pathways Impacted by Stressors
Source: Genes (Basel). 2022 May 2;13(5):814. doi: 10.3390/genes13050814 (PMC9141200; doi:10.3390/genes13050814)
Supplement: Supplementary file 1 [file genes-13-00814-s001.zip › genes-1690593-supplementary.pdf]

Table S1. Log(fold change), False Discovery Rate-adjusted P-value (FDR), and raw P-values of all genes that presented a significant (FDR P-value < 0.05 and |log2(fold change between pig groups)| > 1.2) main effect of maternal immune activation.

Gene Symbol	Fold Change[1]	P-value	FDR
PYURF	-8.58	3.2E-66	5.1E-62
GPR88	-6.01	6.8E-62	5.4E-58
LPL	-4.81	1.2E-43	6.9E-40
PI15	-4.53	8.5E-38	2.5E-34
GPR52	-4.48	1.1E-37	2.5E-34
*DRD2	-4.37	1.0E-37	2.5E-34
C3H16orf89	-4.31	7.3E-36	1.4E-32
*DRD1	-3.95	1.4E-32	2.5E-29
SIX3	-3.98	6.0E-31	9.6E-28
CALB1	-3.90	3.2E-30	4.7E-27
TNMD	-3.80	2.5E-29	3.3E-26
GPR6	-3.76	6.6E-29	8.1E-26
CRABP2	-3.68	7.9E-28	9.0E-25
SLC13A4	-3.46	3.0E-26	3.2E-23
ADORA2A	-3.41	1.0E-25	1.0E-22
SYNDIG1L	-3.34	1.2E-24	1.1E-21
SLC5A5	-3.27	2.4E-23	2.1E-20
*PPP1R1B	-3.14	1.2E-22	1.0E-19
MPZL2	-3.03	3.9E-20	3.1E-17
FOXC2	-3.00	7.7E-20	5.9E-17
POSTN	-2.89	2.1E-19	1.5E-16
KRT19	-2.91	3.0E-19	2.1E-16
AMHR2	3.26	4.2E-19	2.8E-16
TNFRSF11B	-2.92	1.4E-18	9.4E-16
SFRP5	-2.77	1.6E-17	9.3E-15
BNC2	-2.80	2.2E-17	1.2E-14
MME	-2.73	2.2E-17	1.2E-14
WNT8B	3.26	4.6E-17	2.4E-14
*RGS9	-2.62	1.3E-16	6.5E-14
NTS	-2.59	3.0E-16	1.4E-13
FAM83B	-2.82	7.0E-15	3.0E-12
KLF5	-2.57	7.0E-15	3.0E-12
IDO1	-2.60	8.3E-15	3.5E-12
CDH1	-2.48	9.8E-15	4.0E-12
*DRD3	-2.53	1.1E-14	4.5E-12
GPR101	-2.48	3.4E-14	1.3E-11
SLC16A12	-2.43	4.7E-14	1.7E-11
THSD4	-2.38	5.4E-14	1.9E-11
C7	-2.40	8.6E-14	3.0E-11
TBX15	-2.37	3.2E-13	1.1E-10
CRABP1	-2.29	5.5E-13	1.8E-10
ACP7	-2.46	6.2E-13	2.0E-10
ANO3	-2.23	1.0E-12	3.2E-10
TAC1	-2.22	1.3E-12	4.0E-10

RARB	-2.22	1.9E-12	6.0E-10
CRHR2	-2.24	3.4E-12	9.8E-10
ITGA11	-2.22	3.3E-12	9.8E-10
ADAMTSL3	-2.15	8.8E-12	2.5E-9
ANKRD34B	-2.16	1.0E-11	2.8E-9
OTOGL	-2.16	1.0E-11	2.9E-9
*PDE1B	-2.09	1.9E-11	5.3E-9
ADAMTS12	-2.11	4.0E-11	1.0E-8
*SLC5A7	-2.11	4.6E-11	1.1E-8
DSP	-2.07	4.8E-11	1.1E-8
KCTD8	-2.07	5.3E-11	1.3E-8
GPR149	-2.15	5.6E-11	1.3E-8
HTR1B	-2.28	8.4E-11	1.9E-8
HTRA3	-2.04	1.0E-10	2.4E-8
FOXD1	-2.04	1.4E-10	3.2E-8
OSR1	-2.02	1.5E-10	3.4E-8
FMO1	-2.02	1.7E-10	3.8E-8
GJB2	-2.04	1.7E-10	3.8E-8
FOXP2	-2.03	2.1E-10	4.4E-8
TNFRSF8	-2.01	2.5E-10	5.4E-8
GUCY1A3	-1.96	3.0E-10	6.4E-8
SLC2A13	-1.94	4.9E-10	1.0E-7
GXYLT2	-1.99	5.4E-10	1.0E-7
AKAP5	-1.93	6.6E-10	1.3E-7
EGFLAM	-1.95	7.5E-10	1.4E-7
OAZ3	2.14	1.0E-9	2.0E-7
CBLN4	-1.91	1.1E-9	2.2E-7
GPRIN3	-1.89	1.2E-9	2.4E-7
LCN2	-1.90	1.4E-9	2.6E-7
DACH1	-1.89	1.4E-9	2.6E-7
DLX2	-1.91	1.5E-9	2.7E-7
SLC35G1	-1.92	2.2E-9	3.9E-7
HTR2C	-1.86	2.2E-9	3.9E-7
CBLN1	-1.90	2.4E-9	4.2E-7
SV2C	-1.87	2.5E-9	4.3E-7
SLC10A4	-1.89	5.8E-9	9.5E-7
CUX2	-1.81	5.9E-9	9.5E-7
PBX3	-1.81	6.6E-9	1.0E-6
CDCP2	-1.91	7.3E-9	1.1E-6
ITGBL1	-1.82	8.0E-9	1.2E-6
CTSL	2.03	8.5E-9	1.3E-6
CEMIP	-1.79	8.7E-9	1.3E-6
SP9	-1.82	1.0E-8	1.5E-6
BMP6	-1.81	1.1E-8	1.7E-6
UNC13C	-1.76	1.4E-8	2.1E-6
*GNAL	-1.75	1.5E-8	2.2E-6
DGKB	-1.75	1.7E-8	2.5E-6
C1QTNF3	1.93	1.7E-8	2.5E-6
IGSF22	-1.89	2.0E-8	2.9E-6

MEIS2	-1.73	2.2E-8	3.1E-6
STRIP2	-1.73	2.3E-8	3.2E-6
*PDE10A	-1.73	2.5E-8	3.5E-6
HGF	-1.84	2.8E-8	3.8E-6
TPH2	-1.86	2.8E-8	3.9E-6
FN1	-1.71	3.4E-8	4.6E-6
IGF2	-1.71	3.7E-8	4.9E-6
CHRNE	-1.86	3.9E-8	5.1E-6
INPP5F	-1.70	3.9E-8	5.1E-6
RASD2	-1.70	3.9E-8	5.1E-6
PLXDC1	-1.72	4.1E-8	5.2E-6
PCDH11X	-1.71	4.2E-8	5.3E-6
PNLIPRP2	-1.81	4.4E-8	5.4E-6
SLC9A3	1.91	5.5E-8	6.7E-6
TF	1.78	6.7E-8	8.1E-6
ARHGAP6	-1.68	8.0E-8	9.6E-6
LTBP2	-1.68	9.3E-8	1.1E-5
MDFIC2	-1.77	1.0E-7	1.2E-5
ANKRD63	-1.64	1.1E-7	1.3E-5
SCUBE3	-1.78	1.3E-7	1.4E-5
GUCY1A1	-1.65	1.3E-7	1.5E-5
NOV	-1.62	1.6E-7	1.8E-5
SEMA3F	-1.64	1.6E-7	1.8E-5
FSTL5	-1.62	1.6E-7	1.8E-5
HAPLN2	1.72	1.7E-7	1.9E-5
RGS17	-1.62	1.9E-7	2.1E-5
PTCHD1	-1.65	1.9E-7	2.1E-5
GRM4	-1.62	2.0E-7	2.1E-5
SLC6A20	-1.60	2.2E-7	2.3E-5
GPC6	-1.62	2.3E-7	2.4E-5
OPRD1	-1.67	2.6E-7	2.8E-5
ARPP21	-1.59	2.8E-7	3.0E-5
ATP8B4	1.86	3.1E-7	3.2E-5
TNFRSF25	1.74	3.1E-7	3.2E-5
*ATP6V1C2	1.68	3.8E-7	3.9E-5
ADAMTS6	-1.58	4.0E-7	4.0E-5
BMP3	-1.62	4.1E-7	4.1E-5
LRRTM3	-1.56	4.5E-7	4.5E-5
SYT6	-1.57	4.7E-7	4.6E-5
*PDE7B	-1.56	4.7E-7	4.6E-5
LOX	-1.63	4.9E-7	4.8E-5
SPATA1	-1.58	5.1E-7	4.9E-5
PCP4	-1.55	5.3E-7	5.1E-5
TNNT2	1.78	5.5E-7	5.2E-5
SLC26A4	-1.63	5.6E-7	5.3E-5
SYNPO2L	1.64	6.0E-7	5.7E-5
KCNK2	-1.54	6.1E-7	5.7E-5
VWC2L	-1.55	7.2E-7	6.7E-5
MGARP	-1.55	7.7E-7	7.0E-5

C1QTNF7	-1.62	7.7E-7	7.1E-5
*GAD2	-1.53	7.8E-7	7.1E-5
ELMO3	-1.62	8.2E-7	7.4E-5
DPY19L2	-1.57	8.7E-7	7.8E-5
PAPPA2	-1.54	8.9E-7	7.9E-5
NPAS4	-1.65	1.0E-6	9.4E-5
ZIC1	-1.51	1.1E-6	9.8E-5
DCLK3	-1.52	1.2E-6	1.0E-4
*SLC17A6	-1.50	1.2E-6	1.1E-4
LRRC17	-1.53	1.3E-6	1.1E-4
DLX1	-1.50	1.3E-6	1.1E-4
SGCZ	-1.57	1.5E-6	1.3E-4
PPP1R14A	1.57	1.6E-6	1.3E-4
NEXN	-1.52	1.6E-6	1.3E-4
TRH	-1.49	1.6E-6	1.3E-4
SCN4B	-1.47	1.9E-6	1.5E-4
KCTD14	1.59	1.9E-6	1.5E-4
RN5-8S	1.55	2.1E-6	1.7E-4
TC2N	-1.75	2.1E-6	1.7E-4
FAP	-1.53	2.2E-6	1.7E-4
KITLG	-1.46	2.4E-6	1.9E-4
LCN9	1.60	2.6E-6	2.0E-4
CHSY3	-1.46	2.7E-6	2.1E-4
FAM178B	1.56	2.8E-6	2.2E-4
P2RY1	-1.45	2.9E-6	2.2E-4
NKX6-2	1.53	3.0E-6	2.3E-4
KCNAB1	-1.44	3.1E-6	2.3E-4
HK2	-1.46	3.3E-6	2.5E-4
MAG	1.51	3.3E-6	2.5E-4
PKD2L1	1.57	3.5E-6	2.7E-4
PNPLA3	1.59	3.6E-6	2.7E-4
*PDE8B	-1.43	3.7E-6	2.8E-4
KLK6	1.56	3.8E-6	2.8E-4
CACNB4	-1.44	3.8E-6	2.8E-4
TTR	1.52	3.8E-6	2.8E-4
VMO1	1.58	4.0E-6	2.9E-4
RYR1	-1.43	4.1E-6	3.0E-4
DCN	-1.41	4.6E-6	3.3E-4
ALDH1A2	-1.44	4.7E-6	3.3E-4
PAX5	-1.49	4.8E-6	3.4E-4
TES	-1.46	4.9E-6	3.5E-4
FBN1	-1.41	5.1E-6	3.6E-4
KCNRG	1.71	5.4E-6	3.7E-4
ZNF883	-1.43	5.5E-6	3.8E-4
COL12A1	-1.41	5.7E-6	3.9E-4
HTR1F	-1.49	6.0E-6	4.2E-4
GJB1	1.48	6.1E-6	4.2E-4
KL	-1.41	6.2E-6	4.2E-4
MBP	1.47	6.3E-6	4.3E-4

HMCN2	-1.48	6.4E-6	4.3E-4
TMEM125	1.47	6.5E-6	4.3E-4
EFHD1	1.47	6.7E-6	4.5E-4
ADGRG6	-1.40	6.7E-6	4.5E-4
COL3A1	-1.39	6.9E-6	4.6E-4
COL8A1	-1.40	7.1E-6	4.7E-4
FNDC9	-1.41	7.5E-6	5.0E-4
AMPD3	-1.39	7.8E-6	5.1E-4
RHBDL2	1.49	7.8E-6	5.1E-4
CCDC88C	-1.38	7.8E-6	5.1E-4
SLC16A6	-1.41	8.3E-6	5.4E-4
DSEL	-1.38	8.5E-6	5.5E-4
ERBB3	1.45	8.5E-6	5.5E-4
MARCO	-1.89	8.6E-6	5.5E-4
LDB2	-1.37	8.7E-6	5.6E-4
ISLR	-1.37	8.8E-6	5.6E-4
HS6ST2	-1.37	9.0E-6	5.7E-4
SLC39A4	1.50	9.1E-6	5.7E-4
FAM163A	-1.39	9.1E-6	5.7E-4
ADAMTS5	-1.43	9.2E-6	5.8E-4
WNT16	-1.40	9.4E-6	5.9E-4
CBLN3	-1.44	9.8E-6	6.1E-4
TKTL1	1.58	1.0E-5	6.2E-4
S100A4	1.66	1.0E-5	6.5E-4
CDC42EP2	1.44	1.1E-5	6.8E-4
SORCS1	-1.36	1.1E-5	6.8E-4
SCARA5	-1.36	1.2E-5	7.3E-4
PLPP2	1.43	1.2E-5	7.3E-4
ARHGEF5	-1.52	1.3E-5	8.1E-4
APOD	1.41	1.3E-5	8.2E-4
PCSK6	1.41	1.4E-5	8.3E-4
NMBR	-1.47	1.4E-5	8.6E-4
PPIP5K2	-1.34	1.4E-5	8.7E-4
GJC2	1.41	1.4E-5	8.7E-4
FAM196B	-1.40	1.5E-5	9.0E-4
GULP1	-1.34	1.6E-5	9.5E-4
UROC1	1.45	1.8E-5	1.0E-3
CNP	1.39	1.9E-5	1.1E-3
PERP	-1.39	2.0E-5	1.1E-3
DLX5	-1.34	2.0E-5	1.1E-3
GALNT6	1.38	2.3E-5	1.3E-3
MUC19	-1.33	2.4E-5	1.3E-3
SCGN	-1.37	2.4E-5	1.4E-3
*CAMK4	-1.30	2.5E-5	1.4E-3
CNTN5	-1.31	2.6E-5	1.4E-3
RXFP1	-1.30	2.6E-5	1.4E-3
METTL27	1.45	2.6E-5	1.4E-3
PRR5L	1.36	2.7E-5	1.5E-3
CENPE	-1.31	2.8E-5	1.5E-3

SGK2	1.36	2.8E-5	1.5E-3
ALDH3B1	1.36	2.8E-5	1.5E-3
SH3TC2	1.36	2.9E-5	1.6E-3
CTXN3	-1.30	3.0E-5	1.6E-3
ADAM12	-1.29	3.2E-5	1.7E-3
CPED1	-1.30	3.2E-5	1.7E-3
MGP	-1.28	3.3E-5	1.7E-3
TMEM200B	-1.32	3.4E-5	1.8E-3
IGSF5	-1.31	3.4E-5	1.8E-3
OPALIN	1.34	3.4E-5	1.8E-3
PROKR2	-1.34	3.5E-5	1.8E-3
CTSC	-1.28	3.6E-5	1.9E-3
NMUR2	1.61	3.7E-5	1.9E-3
KCNH5	-1.32	3.8E-5	1.9E-3
PHLDB2	-1.29	3.8E-5	1.9E-3
*CHRM4	-1.27	3.8E-5	1.9E-3
DGAT2	-1.28	3.9E-5	1.9E-3
ZFP37	-1.27	3.9E-5	1.9E-3
SLC5A11	1.34	3.9E-5	1.9E-3
CMTM5	1.33	4.0E-5	2.0E-3
GJA9	1.48	3.9E-5	2.0E-3
HRH3	-1.27	4.0E-5	2.0E-3
MAL	1.33	4.2E-5	2.1E-3
PADI2	1.32	4.2E-5	2.1E-3
TFPI	-1.39	4.4E-5	2.1E-3
ETNK2	-1.26	4.6E-5	2.3E-3
TRDN	1.43	4.7E-5	2.3E-3
SFRP4	-1.26	4.8E-5	2.3E-3
PAPPA	1.35	5.0E-5	2.4E-3
CYP26A1	-1.26	5.1E-5	2.5E-3
PLEKHB1	1.31	5.1E-5	2.5E-3
LRRN4CL	-1.26	5.2E-5	2.5E-3
EFEMP1	-1.25	5.2E-5	2.5E-3
SFRP1	-1.25	5.2E-5	2.5E-3
RHOG	1.31	5.2E-5	2.5E-3
KLHDC7A	-1.32	5.2E-5	2.5E-3
C1H15orf48	-1.34	5.3E-5	2.5E-3
ADAMTS4	1.31	5.4E-5	2.5E-3
CLCA2	1.31	5.4E-5	2.5E-3
*GABRA4	-1.24	5.5E-5	2.6E-3
CRYAB	1.30	5.8E-5	2.7E-3
CNTN4	-1.24	5.8E-5	2.7E-3
RAB7B	1.30	6.1E-5	2.8E-3
PLLP	1.29	6.2E-5	2.8E-3
OCA2	1.31	6.2E-5	2.8E-3
DLX6	-1.26	6.3E-5	2.9E-3
PEG10	-1.23	6.3E-5	2.9E-3
P2RY2	1.31	6.4E-5	2.9E-3
GPR62	1.30	6.5E-5	3.0E-3

PHLDA3	1.29	6.7E-5	3.0E-3
SPTLC3	-1.24	6.9E-5	3.1E-3
PENK	-1.22	7.1E-5	3.2E-3
ENPP2	1.28	7.1E-5	3.2E-3
RTKN	1.28	7.2E-5	3.2E-3
PRR18	1.28	7.3E-5	3.2E-3
NECAB1	-1.22	7.5E-5	3.3E-3
ST6GALNAC1	-1.37	7.6E-5	3.3E-3
ARHGAP20	-1.22	7.6E-5	3.3E-3
ELOVL1	1.28	7.6E-5	3.3E-3
ERMN	1.27	7.8E-5	3.4E-3
EPOR	1.33	8.0E-5	3.5E-3
EDAR	-1.26	8.1E-5	3.5E-3
SMIM5	1.29	8.1E-5	3.5E-3
NAALADL2	-1.28	8.2E-5	3.5E-3
LYPD1	-1.22	8.2E-5	3.5E-3
HOXD1	1.29	8.5E-5	3.6E-3
INSC	1.30	8.6E-5	3.7E-3
PLAGL1	-1.22	8.8E-5	3.7E-3
MOBP	1.26	9.7E-5	4.1E-3
SLC6A13	-1.20	9.9E-5	4.1E-3
MCTP2	-1.35	1.0E-4	4.2E-3
SERTAD4	-1.20	1.0E-4	4.2E-3
PLA2R1	1.25	1.0E-4	4.4E-3
FA2H	1.25	1.0E-4	4.4E-3
SLC45A3	1.26	1.0E-4	4.5E-3
RSAD2	-1.22	1.1E-4	4.7E-3
TRPC3	-1.24	1.1E-4	4.7E-3
TMEM88B	1.24	1.1E-4	4.7E-3
ANKRD55	-1.23	1.1E-4	4.7E-3
STAP2	1.33	1.2E-4	4.8E-3
SNORC	1.38	1.2E-4	5.1E-3
BCAS1	1.23	1.3E-4	5.2E-3
KRT25	-1.32	1.3E-4	5.3E-3
KLHL34	1.34	1.3E-4	5.4E-3
CA14	1.23	1.4E-4	5.7E-3
CLDN11	1.22	1.5E-4	5.8E-3
PLP1	1.22	1.5E-4	5.9E-3
ITGA2	-1.21	1.5E-4	6.0E-3
S1PR5	1.22	1.5E-4	6.1E-3
HTR6	-1.21	1.6E-4	6.2E-3
LRRC75B	1.25	1.7E-4	6.7E-3
MYMK	1.34	1.7E-4	6.7E-3
RNF212	1.30	1.8E-4	7.0E-3
TCTEX1D4	1.48	1.9E-4	7.1E-3
C14H10orf90	1.20	2.0E-4	7.4E-3
CLIC3	-1.20	2.0E-4	7.6E-3
CARD11	1.22	2.0E-4	7.7E-3
TTLL6	1.26	2.2E-4	8.1E-3

DMRT2	-1.24	2.3E-4	8.5E-3
MPL	1.30	3.4E-4	1.1E-2
S100A3	1.23	3.9E-4	1.3E-2
TCF15	1.20	4.1E-4	1.3E-2
AGMO	1.31	4.3E-4	1.4E-3
SNTN	1.25	6.1E-4	1.9E-2
ADGRG3	-1.26	6.3E-4	1.9E-2
OC90	1.23	6.3E-3	1.9E-2
KIF12	1.20	7.5E-4	2.2E-2

[1]MIA = maternal immune activation effect where NES > 0 (NES < 0) denotes gene over-expression (under-expression) in Control relative to MIA pigs

* = gene is present in an enriched pathway indicated in Table 1

Table S2. Log(fold change), False Discovery Rate-adjusted P-value (FDR), and raw P-values of all genes that presented a significant (FDR P-value < 0.05 and |log2(fold change between pig groups)| > 1.2) main effect of weaning.

Gene Symbol	Fold Change[1]	P-value	FDR
PYURF	8.71	2.0E-69	3.3E-65
WNT8B	-3.06	3.1E-17	1.2E-13
ACP7	2.33	2.7E-12	8.6E-9
GH1	-2.65	1.1E-11	2.6E-8
C1QTNF3	-1.88	4.9E-9	8.8E-6
OTX2	-2.06	1.8E-8	3.0E-5
CCDC42	1.77	4.1E-8	5.4E-5
GBP1	-1.64	1.2E-7	1.4E-4
ATP5PD	-1.61	1.8E-7	2.0E-4
*SLA-DRA	-1.67	2.8E-7	2.8E-4
SIX3	-2.06	3.3E-7	3.0E-4
ABCA6	1.62	3.2E-7	3.0E-4
CRABP1	-1.65	4.2E-7	3.4E-4
ST6GALNAC1	1.70	4.3E-7	3.4E-4
*CTSL	-1.62	5.9E-7	4.3E-4
COL1A1	-1.52	6.6E-7	4.6E-4
WNT2B	-1.67	1.2E-6	8.3E-4
*SLA-DQA1	-1.56	2.6E-6	1.4E-3
UBD	-2.31	3.0E-6	1.6E-3
LCN2	-1.49	3.4E-6	1.7E-3
GPR101	-1.58	1.2E-5	5.7E-3
KLK6	-1.35	2.1E-5	9.4E-3
TREX2	1.34	2.1E-5	9.4E-3
MLPH	1.31	2.4E-5	1.0E-2
ARG1	-1.54	2.5E-5	1.0E-2
ANGPTL4	1.27	2.6E-5	1.1E-2
TMC2	-1.60	3.2E-5	1.2E-2
NUPR1	1.24	4.3E-5	1.6E-2
CHST13	-1.48	4.7E-5	1.7E-2
CXCL10	-1.34	5.0E-5	1.8E-2
ALDH1A2	-1.30	5.3E-5	1.9E-2
KCNG4	-1.27	6.2E-5	2.1E-2
C1QTNF6	-1.24	7.2E-5	2.3E-2
CNMD	-1.22	7.5E-5	2.4E-2
SLC30A8	-1.37	1.0E-4	3.2E-2
AIRE	1.22	1.3E-4	3.8E-2

[1]Wean = weaning effect where NES > 0 (NES < 0) denotes gene over-expression (under-expression) in weaned relative to nursed pigs.

* = gene is present in an enriched pathway indicated in Table 1

Table S3. Log(fold change), False Discovery Rate-adjusted P-value (FDR), and raw P-values of all genes that presented a significant (FDR P-value < 0.05 and ∣log2(fold change between pig groups)∣ > 1.2) main effect of sex.

Gene Symbol	Fold Change[1]	P-value	FDR
EIF1AY	12.85	7.1E-99	2.2E-96
PYURF	7.99	6.4E-59	1.0E-55
GPR88	5.69	1.5E-58	2.2E-55
DRD2	4.57	8.5E-42	9.0E-39
SYNDIG1L	3.81	8.6E-32	8.6E-29
SIX3	3.95	1.2E-31	1.1E-28
ADORA2A	3.77	2.2E-31	2.0E-28
LPL	3.47	1.2E-26	1.0E-23
PPP1R1B	3.24	9.5E-25	7.6E-22
DRD1	3.25	1.2E-24	9.5E-22
CALB1	3.21	1.2E-22	9.0E-20
GPR52	3.10	4.0E-21	2.7E-18
CHRNE	3.15	3.9E-21	2.7E-18
S100A4	3.01	2.0E-20	1.2E-17
GPR6	2.79	3.0E-18	1.7E-15
WNT8B	-2.94	2.4E-16	1.4E-13
RGS9	2.42	2.9E-15	1.6E-12
TPH2	2.54	7.8E-15	4.2E-12
CRABP1	2.25	2.9E-13	1.5E-10
TAC1	1.96	8.9E-11	4.0E-8
WNT2B	-2.21	3.9E-10	1.7E-7
SLC10A4	1.93	8.3E-10	3.6E-7
PDE1B	1.74	5.8E-9	2.4E-6
DRD3	1.77	1.8E-8	7.3E-6
C1QTNF3	-1.78	2.6E-8	1.0E-5
TMC2	-2.23	2.7E-8	1.0E-5
ANKRD63	1.66	3.0E-8	1.1E-5
PCP4	1.65	3.5E-8	1.3E-5
TNFRSF8	1.66	6.3E-8	2.1E-5
RASD2	1.57	1.4E-7	4.5E-5
OTOGL	1.59	1.6E-7	5.2E-5
RARB	1.55	2.5E-7	7.9E-5
KCNG4	-1.64	3.6E-7	1.1E-4
MME	1.54	4.3E-7	1.3E-4
GABRE	-1.66	6.9E-7	2.0E-4
ANO3	1.47	7.9E-7	2.2E-4
GNAL	1.46	8.8E-7	2.4E-4
*CTSL	-1.56	1.3E-6	3.5E-4
DGAT2	1.45	1.3E-6	3.5E-4
CDCP2	1.54	1.3E-6	3.5E-4

SLC5A7	1.49	1.4E-6	3.6E-4
CCDC17	-1.66	1.6E-6	4.1E-4
NTS	1.43	1.7E-6	4.3E-4
OAZ3	-1.50	2.2E-6	5.6E-4
NEXN	1.42	3.2E-6	7.8E-4
ITGA11	1.41	3.8E-6	9.0E-4
MGARP	1.38	5.1E-6	1.1E-3
TMEM200B	1.40	5.4E-6	1.2E-3
*SLA-DRA	-1.44	5.6E-6	1.2E-3
PENK	1.35	5.8E-6	1.2E-3
ELMO3	1.42	7.4E-6	1.5E-3
TMIE	-1.39	8.1E-6	1.6E-3
CARD11	-1.39	8.5E-6	1.7E-3
KCTD8	1.34	8.9E-6	1.7E-3
SP9	1.35	9.5E-6	1.8E-3
PTCHD1	1.34	1.1E-5	2.1E-3
MEIS2	1.30	1.1E-5	2.1E-3
TTR	-1.34	1.2E-5	2.3E-3
BICDL2	1.40	1.2E-5	2.3E-3
DLX2	1.32	1.3E-5	2.4E-3
PBX3	1.30	1.4E-5	2.6E-3
TRDN	-1.43	1.6E-5	3.0E-3
SCN4B	1.28	1.7E-5	3.0E-3
MYO15A	1.29	1.9E-5	3.4E-3
ADRA2C	1.27	1.9E-5	3.4E-3
RYR1	1.28	1.9E-5	3.4E-3
CPS1	-1.41	2.2E-5	3.9E-3
PLXDC1	1.26	2.7E-5	4.5E-3
TRIM63	-1.31	2.7E-5	4.6E-3
FREM1	-1.31	2.9E-5	4.9E-3
TNFRSF25	-1.31	3.1E-5	5.1E-3
KL	1.25	3.4E-5	5.5E-3
IGSF22	1.36	3.5E-5	5.6E-3
TIGIT	-1.26	4.8E-5	7.6E-3
*HMGCS2	-1.37	6.4E-5	1.0E-2
ISLR	-1.22	6.9E-5	1.0E-2
SLC25A48	1.20	8.4E-5	1.2E-2
WFIKKN2	-1.24	9.7E-5	1.4E-2
KRT5	1.25	1.0E-4	1.4E-2
CXCL10	-1.25	1.1E-4	1.6E-2
BHLHE23	-1.39	1.6E-4	2.1E-2
RIPPLY3	-1.28	1.7E-4	2.2E-2
SLC5A5	-1.24	1.8E-4	2.3E-2
DMRT2	-1.40	2.0E-4	2.5E-2
PRLHR	-1.29	3.0E-4	3.5E-2

GH1	-1.22	3.6E-4	4.0E-2
CHST13	-1.24	4.4E-4	4.8E-2

[1]Sex effect where NES > 0 (NES < 0) denotes gene over-expression (under-expression) in males relative of females

* = gene is present in an enriched pathway indicated in Table 1

Table S4. Log(fold change) of pairwise contrasts of genes in the terpenoid backbone biosynthesis pathway.

Gene	CNF[1]	PNF	CWF	PWF	PNM	CWM	PWM
ACAT1	0.02	-0.03	-0.09	-0.06	-0.09	-0.07	-0.12
ACAT2	0.17	-0.49	-0.18	-0.69	0.15	-0.08	-0.12
DHDDS	-0.02	0.07	-0.24	-0.02	0.18	0.10	0.11
FDPS	0.26	-0.49	0.03	-0.84	-0.11	-0.25	-0.25
FNTA	0.31	-0.58	0.08	-0.46	0.20	0.41	0.16
FNTB	0.11	-0.33	-0.01	-0.35	-0.03	-0.03	-0.05
HMGCR	0.21	-0.28	-0.39	-0.37	0.60	0.35	0.27
HMGCS1	0.18	-0.27	-0.36	-0.55	0.42	0.28	0.05
HMGCS2	1.38	0.95	1.01	2.00	1.18	1.76	1.01
MVD	0.37	-0.44	0.14	-0.75	-0.13	-0.31	-0.30
MVK	0.26	-0.53	-0.22	-0.95	-0.10	-0.25	-0.41
PCYOX1	-0.04	0.07	-0.21	0.04	0.31	0.35	0.21
PDSS1	-0.36	0.02	-0.47	-0.19	-0.03	-0.05	-0.29
PMVK	0.31	-0.34	0.19	-0.51	-0.11	-0.21	-0.04

[1]CNF = Control nursed females, PNF = PRRSV nursed females, CWF = Control weaned females, PWF = PRRSV weaned females, PNM = PRRSV nursed males, CWM = Control weaned males, PWM = PRRSV weaned males

Table S5. Log(fold change) of pairwise contrasts of genes in the cocaine addiction pathway.

Gene	CNF[1]	CWF	CWM	PNF	PNM	PWF	PWM
ADCY5	-0.84	0.10	-0.80	-1.21	-0.99	-0.96	-1.00
ATF2	-0.21	0.43	0.02	0.41	0.45	0.61	0.53
CDK5R1	-0.33	0.19	-0.03	0.12	-0.12	-0.10	0.01
CREB1	-0.12	0.36	-0.10	0.20	0.35	0.56	0.39
DRD1	-3.26	0.70	-2.67	-2.84	-2.94	-2.68	-2.69
DRD2	-4.58	-0.21	-5.50	-6.29	-5.62	-5.96	-5.85
GRIA2	-0.33	0.33	0.04	0.46	0.32	0.74	0.52
GRIN2C	0.14	0.57	0.32	0.09	0.13	0.22	-0.09
GRM3	-0.33	0.13	-0.28	-0.35	-0.21	-0.27	-0.13
PPP1R1B	-3.24	-0.09	-3.17	-3.78	-3.89	-3.59	-3.86
PRKACB	-0.20	0.52	-0.17	0.29	0.21	0.27	0.26
RGS9	-2.43	0.20	-2.45	-2.76	-2.85	-2.91	-2.97

[1]CNF = Control nursed females, PNF = PRRSV nursed females, CWF = Control weaned females, PWF = PRRSV weaned females, PNM = PRRSV nursed males, CWM = Control weaned males, PWM = PRRSV weaned males
